# Burden of diarrheal diseases among children under five in East Africa, 2000–2023: insights from the global burden of disease 2023 study

**DOI:** 10.1186/s12879-026-13344-0

**Published:** 2026-04-22

**Authors:** Hiba Abdi Salad, Sharmake Gaiye Bashir

**Affiliations:** 1 Faculty of Health Sciences and Tropical Medicine, Somali National University, Mogadishu, Somalia; 2 Faculty of Health Science, Salaam University, Mogadishu, Somalia; 3 Center for Health Research and Innovation, Somali National University, Mogadishu, Somalia

**Keywords:** Diarrhoeal disease, Under-five mortality, East Africa, Global Burden of Disease, WASH, Health equity, SDG 3.2

## Abstract

**Background:**

Diarrheal disease remains a leading cause of under-five mortality in East Africa. However, comprehensive contemporary comparative analyses across countries remain limited. This study quantifies the disease burden and temporal trends using the Global Burden of Disease (GBD) 2023 estimates.

**Methods:**

We extracted the incidence, prevalence, mortality, and disability-adjusted life-years (DALYs) for diarrheal diseases among children under five across 12 East African countries (2000–2023) from the GBD Results Tool. We analyzed the temporal trends, country-level variations, and DALYs attributable to water, sanitation, and hygiene (WASH) risk factors using descriptive analysis and trend visualization.

**Results:**

Mortality declined by 60–87% in high-performing countries (Tanzania, 84%; Rwanda, 88%; Uganda, 82%; Ethiopia, 86%), whereas Somalia, South Sudan, and Madagascar demonstrated minimal improvement (14.7–69.2% decline), representing a 12-fold difference in 2023 mortality rates (Tanzania 36 vs. South Sudan 443 per 100,000). Corresponding DALY reductions ranged from 80 to 87% in improving countries to 15–69% in countries with stalled progress. Age-stratified analysis revealed that neonates experienced minimal decline (15%) compared with older children (60–80%). Males sustained 25–36% higher mortality and DALYs despite convergent incidence and prevalence. The WASH-attributable DALYs ranged from 3,057 per 100,000 (Tanzania) to 37,608 per 100,000 (South Sudan), with unsafe water contributing 70–80% of the burden.

**Conclusions:**

East Africa achieved substantial but profoundly inequitable reduction in diarrheal disease mortality. Achieving SDG 3.2 requires equitable WASH investment, health system strengthening in conflict-affected regions, and integrated multisectoral approaches addressing water access, sanitation, nutrition, and healthcare delivery simultaneously.

**Clinical trial number:**

Not applicable.

## Introduction

Diarrheal diseases remain a leading cause of morbidity and mortality among children under five years of age globally, despite decades of public health intervention and medical advancement [1]. Globally in 2021, diarrhoeal diseases caused an estimated 1.17 million deaths (95% uncertainty interval 0.793–1.62 million), representing approximately 60% reduction from 1990, yet this progress masks profound persistent inequities in disease burden across geographical regions and socioeconomic strata [2]. While mortality rates have declined in many high-income settings through improved water, sanitation, and hygiene infrastructure alongside the widespread implementation of effective interventions such as oral rehydration therapy and rotavirus vaccination, children in low- and middle-income countries continue to bear a disproportionate share of diarrheal disease mortality and disability [3–5]. Among children under five years, diarrheal diseases account for approximately one in ten deaths worldwide, with the disease concentrated heavily in Sub-Saharan Africa and South Asia, which together accounted for approximately 78% of all diarrhea deaths among this age group [3, 5]. The substantial remaining mortality burden underscores persistent gaps in both access to prevention and treatment, as well as an insufficient understanding of regional disease epidemiology and evolving risk factor contributions [3].

Sub-Saharan Africa remains disproportionately affected by diarrheal disease burden among children under five years [6]. Sub-Saharan Africa accounted for over 90% of the loss of life from diarrhea in infants and young children in developing nations, with the disease consistently ranking among the top five causes of under-five mortality across the region [6]. Within this broader regional context, East Africa experiences particular vulnerability, with a high diarrheal disease burden concentrated in countries including Ethiopia, Kenya, Uganda, Tanzania, and Somalia, characterized by limited healthcare system capacity, widespread poverty, and constrained access to basic water and sanitation infrastructure [7]. Recent systematic reviews examining diarrhea among under-five children in East Africa between 2013 and 2023 have documented high prevalence rates with marked geographic and seasonal variation, ranging from 11% to 54% across different settings and time periods, along with substantial associations with multiple socioeconomic and behavioral determinants [4]. However, comparative, population-level analyses integrating data across East African countries and quantifying time trends in incidence, mortality, and disability burden over extended periods remain limited, particularly when using standardized methodology that permits cross-country evaluation [8].

Water, sanitation, and hygiene deficiency are foundational determinants of diarrheal disease burden in East Africa, accounting for a substantial proportion of attributable diseases and preventable deaths among children under five years of age [9, 10]. Access to improved drinking water sources and sanitation facilities remains inadequate across much of East Africa, with unimproved water sources and unsafe sanitation identified as contributing factors to 72.1% and significant proportions of the diarrhea burden, respectively [10, 11]. Recent spatial analyses of the East African region have revealed substantial clustering of unimproved drinking water sources across 12 East African countries, with 68.7% of water at very high risk derived from unimproved sources, including unprotected wells, surface water, and vendor-provided supplies [12]. Diarrheal disease risk factors operate within households and communities with poor sanitation infrastructure. Unsafe disposal of human waste remains prevalent, particularly in rural and informal urban settlements where defecation in the open and use of unimproved latrines continue despite sustained advocacy for improved practices [13, 14]. Additionally, hand hygiene practices remain suboptimal in many households, with delayed breastfeeding initiation, low vitamin A supplementation coverage, and delayed introduction of appropriate complementary feeding contributing to heightened child vulnerability [15]. The combined burden attributable to WASH factors and nutritional determinants remains substantial and largely preventable through targeted investment and behavioral change [9, 10, 13].

Malnutrition serves as a critical cofactor amplifying diarrheal disease severity, duration, and adverse outcomes among East African children under five [16]. Wasting (low weight-for-height score) emerged as a leading underlying risk factor for diarrhea mortality, accounting for 80.4% (95% uncertainty interval 68.2–85.0%) of the attributable diarrhea burden [17]. The bidirectional relationship between diarrhea and malnutrition creates a vicious cycle in which acute diarrheal episodes precipitate nutrient malabsorption and growth faltering, while existing protein-energy malnutrition impairs intestinal integrity and immune competence, increasing susceptibility to enteric pathogens and prolonging disease duration [18]. Persistent diarrhea, defined as episodes lasting 14 days or longer, accounts for substantial mortality among undernourished children, with over 30% of diarrheal deaths in Ethiopia, Uganda, and Tanzania occurring in children with concurrent malnutrition [19, 20]. The co-occurrence of malnutrition and diarrheal disease indicates that interventions addressing a single determinant will likely be insufficient; rather, integrated approaches addressing nutrition, food security, and enteric pathogen control are essential for meaningful mortality reduction [21, 22].

Health system fragility in East Africa substantially impairs the delivery of essential diarrheal disease prevention and management services, contributing to excess mortality, despite the availability of evidence-based interventions [4, 20]. Many East African countries operate under resource constraints, characterized by inadequate infrastructure, limited human resources, weak disease surveillance systems, and fragmented financing mechanisms [20, 23]. Healthcare utilization for childhood diarrhea remains low in many settings, with delays in care-seeking and limited access to quality facility-based management contributing to preventable deaths [4, 24]. Provider capacity for appropriate case management, encompassing correct assessment of dehydration severity, appropriate fluid replacement with oral or intravenous rehydration therapy, and recognition of complications requiring antimicrobial therapy, varies substantially across public and private facilities [3, 25]. Furthermore, supply chain disruptions affect the availability of essential medicines and supplies, such as oral rehydration salts, zinc supplementation, and antibiotics for severe diarrhea [20]. The fragility of health systems in the region has been further exposed during disease outbreaks and humanitarian crises, with cholera and other waterborne diarrheal diseases periodically overwhelming the facility capacity [20, 26].

Climate change and recurrent humanitarian crises in East Africa exacerbate the burden of diarrheal disease through multiple pathways affecting food security, water access, and population health vulnerability [27, 28]. East Africa, particularly the Horn of Africa extending from Somalia and Eritrea through Ethiopia and Kenya, represents one of the world’s most climate-vulnerable regions, experiencing the devastating convergence of droughts, floods, food insecurity, and armed conflict that undermines livelihood strategies and health system functioning [29, 30]. Between 2030 and 2050, climate change is projected to cause an additional 250,000 deaths annually due to infectious diseases, including diarrhea and heat stress, in low-income countries, with African populations bearing disproportionate impacts [31, 32].

Progress toward Sustainable Development Goal 3.2—ending preventable deaths of children under five—remains substantially off-track in East Africa, with diarrheal disease constituting a persistent barrier to achieving the target of 25 deaths per 1,000 live births by 2030 [33–35]. While global under-five mortality has declined substantially from 1990 to 2021 and many East African countries have achieved reductions in child mortality rates, the pace of decline remains insufficient to meet SDG targets [34, 36, 37]. Only eight of 54 African countries have achieved an under-five mortality rate of 25 deaths per 1,000 live births or lower, and many face stalled progress or reversed gains [35]. Persistent knowledge gaps regarding the contemporary epidemiology and burden of diarrheal diseases in East Africa have limited evidence-based planning and resource allocation. While global and regional estimates of diarrheal disease burden have been published, systematic comparative analyses quantifying disease trends across multiple East African countries using standardized methodology over extended periods, with particular attention to age-, sex-, and spatiotemporal variation, are limited [7, 38, 39]. Furthermore, few studies have integrated the analysis of diarrheal disease burden with comprehensive characterization of region-specific risk factor contributions, particularly WASH-related determinants and their modification in different geographic and social contexts across East Africa [7].

This study used data from the Global Burden of Disease 2023 to quantify the incidence, prevalence, mortality, and disability-adjusted life years (DALYs) attributable to diarrheal diseases among children under five years of age in East Africa from 2000 to 2023. It further examined temporal trends, country-level variations, and the contribution of WASH-related risk factors, providing updated evidence to inform policy, intervention planning, and progress toward SDG 3.2.

## Methodology

### Study design and data source

This study is a secondary analysis of Global Burden of Disease (GBD) 2023 estimates describing the incidence, prevalence, mortality, and disability-adjusted life years (DALYs) associated with diarrheal diseases among children under five years of age in East Africa over the period 2000–2023. The GBD 2023 study is a comprehensive, systematic analysis that integrates multiple sources, including household surveys, demographic and health surveys, vital registration systems, disease surveillance data, and hospital records, using standardized, reproducible methodology to generate modeled estimates of disease burden.

### Study population and scope

We included 12 East African countries, as defined by the World Health Organization (WHO): Burundi, Comoros, Djibouti, Eritrea, Ethiopia, Kenya, Madagascar, Rwanda, Somalia, South Sudan, Uganda, and the United Republic of Tanzania. The target population was children under five years of age (< 5 years), reflecting the greatest demographic risk for diarrheal diseases in this region.

### Data extraction and GBD 2023 modeling framework

Estimates of the incidence and prevalence of diarrheal diseases were derived using DisMod-MR 2.1, which is a Bayesian mixed-effects meta-regression modeling framework specifically designed to synthesize disparate data sources, address inconsistencies, and correct for systematic biases in epidemiological estimates. DisMod-MR 2.1 uses a compartmental epidemiological model, employs hierarchical meta-regression and borrowing strength across geographies and years, especially benefiting settings with sparse data.

DALYs in the GBD 2023 framework were computed as the sum of Years of Life Lost (YLLs) due to premature mortality and Years Lived with Disability (YLDs) due to a non-fatal illness. Both YLLs and YLDs were modeled within the DisMod-MR 2.1 meta-regression framework, to ensure internal consistency across incidence, prevalence, and mortality estimates.

The cause of Death Ensemble Model (CODEm) was used to estimate cause-specific mortality by combining multiple predictive models and weighting them based on the out-of-sample predictive validity.

For each country and year, 95% uncertainty intervals (UIs) were calculated in DisMod-MR 2.1 framework using a probabilistic approach with 1,000 draws from the posterior distribution of each parameter, thus reflecting both data input and model uncertainty.

All estimates for East African countries were accessed via the GBD 2023 Results Tool (https://www.healthdata.org/research-analysis/about-gbd), and metadata were obtained from the GBD 2023 Sources Tool (https://www.healthdata.org/research-analysis/about-gbd).

### Outcomes and variables

The primary outcomes were:

- Incidence rate: diarrhea incidence per 100,000 children under five.
- Prevalence rate: diarrhea prevalence per 100,000 children under five.
- Mortality rate: diarrhea death rate per 100,000 children under five.
- DALY rate: total burden (YLL + YLD) per 100,000 children under five.

All estimates were reported with their corresponding 95% UIs calculated using posterior distributions from DisMod-MR 2.1.

### Risk factors

To assess the determinants of diarrheal disease burden, this study examined risk-attributable DALYs for key water, sanitation, and hygiene (WASH) exposure included in the GBD 2023 framework. These comprised *unsafe water sources*,* unsafe sanitation*,* and lack of handwashing facilities.*

Attributable DALYs represent the proportion of the total disease burden causally linked to each exposure, estimated using the GBD comparative risk assessment framework. This approach integrates exposure data, relative risks from meta-analyses, and population-attributable fractions to quantify the contribution of each risk factor to the total diarrheal burden among children under five years of age in East Africa.

### Statistical analysis

#### Descriptive summaries

Extracted estimates were first compiled and cleaned in Microsoft Excel and subsequently analyzed using R version 4.3.2 (R Foundation for Statistical Computing, Vienna, Austria). Descriptive statistics were employed to summarize the incidence, prevalence, and mortality rates of diarrheal diseases among children under five years of age across 12 East African countries using 2023 estimates from the Global Burden of Disease (GBD) study, while temporal trends were analyzed over the full period from 2000 to 2023.

#### Calculation of changes and statistical significance

The percentage change (PC) between 2000 and 2023 was calculated for each indicator and country, using the following formula:

$$\:PC=\frac{(Estimat{e}_{2000}-Estimat{e}_{2023})}{Estimat{e}_{2000}}\times\:100$$

where $\:Estimat{e}_{2000}$ and $\:Estimat{e}_{2023}$ represent the GBD point estimates for each respective year.

To evaluate the statistical significance of the observed changes, the 95% uncertainty intervals (UIs) were compared across years. Non-overlapping UIs were interpreted as statistically significant differences between 2000 and 2023.

#### Trend and visualization

An exploratory trend analysis was conducted using log-transformed estimates to evaluate the direction and magnitude of changes in the diarrheal disease burden from 2000 to 2023. Trends were examined for incidence, prevalence, mortality, and DALY rates among children under five years of age across 12 East African countries.

Visualization and statistical graphics were generated in R version 4.3.2, using the ggplot2 and sf packages to produce multi-panel time-series plots and choropleth maps, illustrating both temporal trends and spatial heterogeneity in the disease burden and risk-attributable DALYs.

## Results

Table 1 shows that from 2000 to 2023, diarrheal disease mortality in East Africa declined substantially in most countries, with Tanzania achieving the greatest reduction at 84.1% (2023 rate: 36.0 per 100,000; 95% UI 23.6–51.8), followed by Rwanda (87.8% decline to 67.9 per 100,000; 95% UI 34.8–115.1) and Uganda (82.1% decline to 40.6 per 100,000; 95% UI 18.0–76.0). However, three countries—South Sudan, Somalia, and Madagascar—demonstrated substantially slower progress, with South Sudan showing minimal improvement of only a 14.7% decline (2023 rate: 443.2 per 100,000; 95% UI 254.4–668.3), Somalia declining 69.2% to 320.5 per 100,000 (95% UI 187.6–488.1), and Madagascar declining 66.5% to 191.5 per 100,000 (95% UI 113.1–288.9). Correspondingly, disability-adjusted life-years (DALYs) followed parallel patterns, declining by 83.9–87.8% in best-performing countries (Tanzania: 3,310 DALYs per 100,000; Uganda: 3,720 DALYs per 100,000; Rwanda: 6,170 DALYs per 100,000) compared to 15.4–69.2% in countries with slower progress (South Sudan: 39,659 DALYs per 100,000; Somalia: 28,724 DALYs per 100,000). The prevalence declined by 60–79% across most countries, while incidence reductions ranged from 51 to 75%, with a convergence of 63,000–115,000 cases per 100,000 in 2023 across improving countries. South Sudan and Somalia maintained incidence rates of 88,700–153,200 per 100,000, reflecting persistent transmission in conflict-affected and climate-vulnerable regions.

**Table 1:** Epidemiological indicators of diarrheal disease burden in East African Countries per 100,000 (2000–2023)

location	Deaths_2023 (95% UI)	Deaths_% change 2000–2023	DALYs_2023 (95% UI)	DALYs_% change 2000–2023_	Prevalence_2023 (95% UI)	Prevalence_% change 2000–2023	Incidence_2023 (95% UI)	Incidence_% change 2000–2023
Burundi	104.1 (47.0–185.8)	-79.4869	9354.6 (4305.0–16576.5)	-79.4934	1048.6 (894.5–1209.0)	-79.0393	72453.3 (60776.6–84378.9)	-74.0016
Comoros	98.1 (49.5–159.5)	-78.2724	8854.3 (4501.1–14310.6)	-78.266	1067.7 (929.0–1224.7)	-76.5125	73080.0 (62717.5–85404.8)	-72.0898
Djibouti	118.9 (63.9–192.6)	-76.6508	10790.1 (5863.8–17340.6)	-76.5251	1745.8 (1511.9–2041.0)	-66.468	115579.4 (98533.2–136173.5)	-59.3407
Eritrea	131.1 (70.9–216.0)	-71.4232	11753.1 (6405.4–19221.4)	-71.4347	1273.1 (1094.9–1472.9)	-74.1313	87299.3 (73372.9–103599.2)	-68.7274
Ethiopia	54.0 (29.2–93.4)	-85.7654	4928.0 (2732.6–8448.6)	-85.5858	1133.7 (947.6–1350.6)	-77.121	75664.9 (61148.6–90891.3)	-73.9733
Kenya	77.4 (53.9–112.3)	-80.4001	7081.6 (4958.3–10228.4)	-80.1347	1714.9 (1447.7–2045.8)	-61.3006	109013.7 (89161.1–130928.3)	-58.1885
Madagascar	191.5 (113.1–288.9)	-66.5029	17222.7 (10291.6–25786.4)	-66.4476	2238.2 (1981.5–2574.3)	-60.3686	147431.2 (126903.8–172860.3)	-51.2916
Rwanda	67.9 (34.8–115.1)	-87.8126	6170.4 (3238.5–10317.1)	-87.6661	1220.0 (1064.2–1411.8)	-75.7503	82955.4 (71308.6–96349.2)	-70.1291
Somalia	320.5 (187.6–488.1)	-69.1936	28724.5 (16854.0–43650.9)	-69.2016	1330.7 (1159.0–1527.8)	-72.7592	88736.5 (75546.0–102739.7)	-68.1378
South Sudan	443.2 (254.4–668.3)	-14.6988	39659.3 (22812.4–59699.4)	-15.374	2417.1 (2139.4–2747.2)	-59.2226	153222.1 (130674.6–177029.3)	-52.3994
Uganda	40.6 (18.0–76.0)	-82.1303	3720.0 (1687.4–6883.9)	-82.0727	993.7 (848.8–1162.8)	-78.7846	68530.9 (56767.6–82179.3)	-74.7052
United Republic of Tanzania	36.0 (23.6–51.8)	-84.099	3310.0 (2200.5–4721.7)	-83.9609	918.6 (790.3–1053.6)	-77.5666	63226.0 (53720.9–73862.4)	-73.0442

**Fig. 1:**
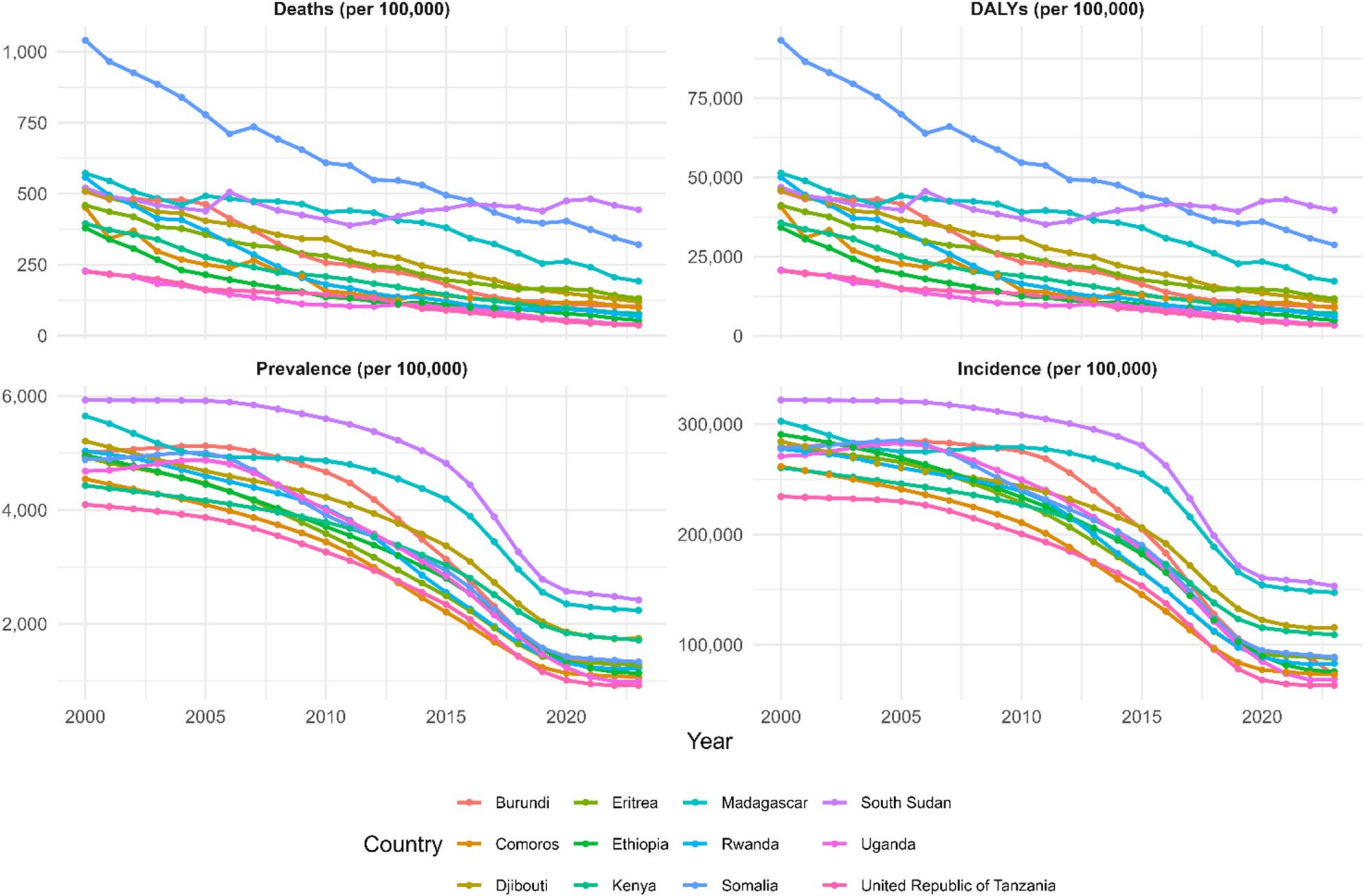
Temporal trends in diarrhoeal disease under 5 years old metrics across East African countries (2000–2023)

Between 2000 and 2023, diarrheal disease mortality declined from approximately 1,000 per 100,000 to 100–500 per 100,000 in improving countries (Uganda, Rwanda, Tanzania, Kenya, and Ethiopia), representing 60–80% reductions, whereas Somalia, South Sudan, and Madagascar remained elevated at 400–1,000 per 100,000, with minimal decline. Correspondingly, DALYs decreased from 100,000 per 100,000 to 10,000–40,000 per 100,000 in countries achieving substantial progress (50–70% reduction), whereas Somalia, South Sudan, and Madagascar sustained DALYs of 35,000–105,000 per 100,000. The prevalence declined from 4,000 to 6,000 per 100,000 to 1,000–2,500 per 100,000 in improving countries (55–75% reduction), and incidence fell from 250,000 to 300,000 per 100,000 to 50,000–150,000 per 100,000 (50–70% decline), whereas countries with stalled progress maintained a prevalence of 3,500–6,000 per 100,000 and an incidence of 150,000–300,000 per 100,000 throughout the period, as shown in Fig. 1.

**Fig. 2:**
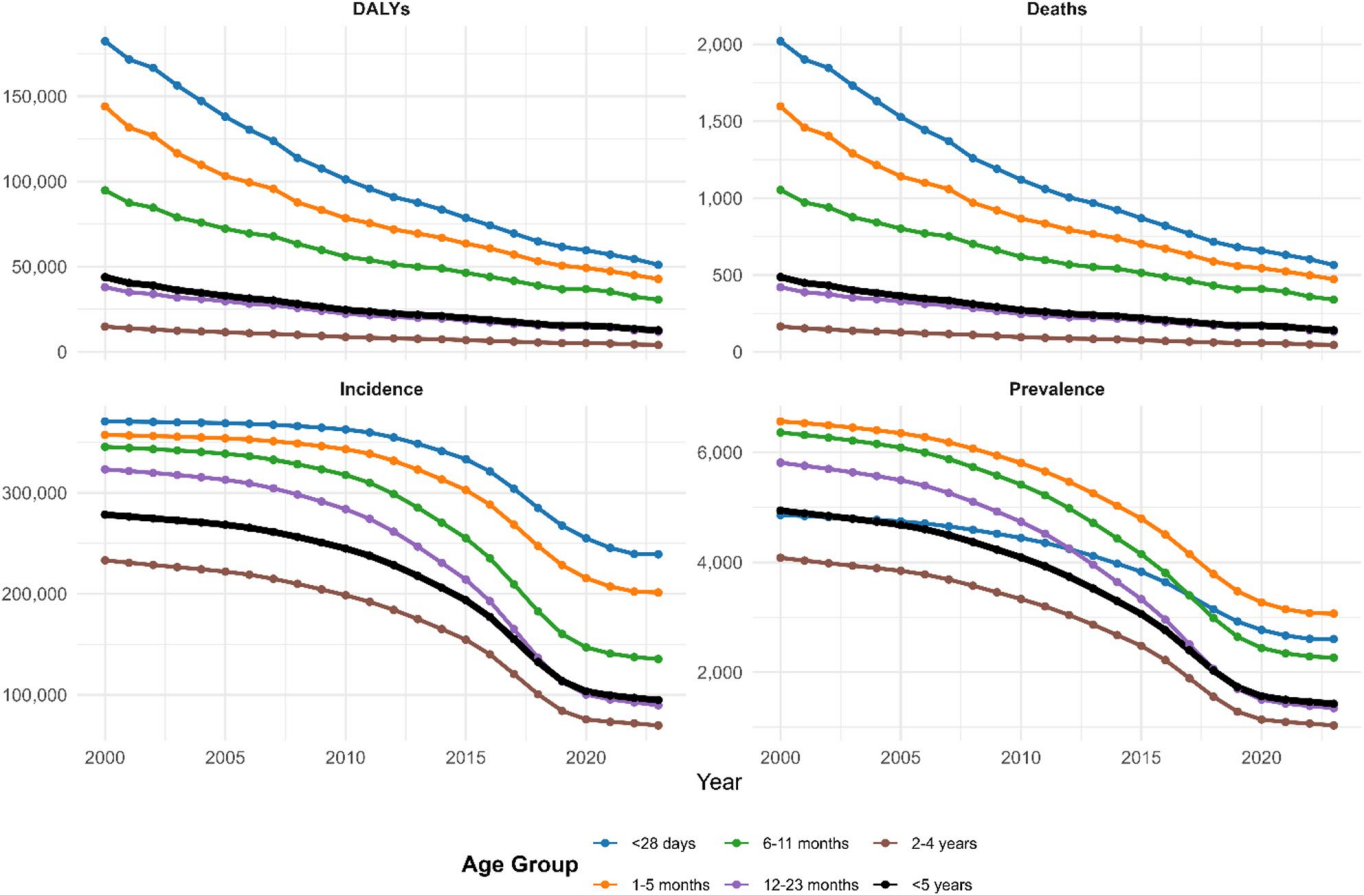
Age-specific trends in diarrhoeal disease burden among children under five in East Africa, 2000–2023

Across all four epidemiological metrics, diarrheal disease burden demonstrated pronounced age-dependent patterns, with infants aged less than 28 days bearing the highest absolute burden. In 2023, children aged less than 28 days experienced 170,000 DALYs per 100,000 and approximately 2,000 deaths per 100,000, declining from 2000 estimates of 200,000 DALYs and 2,200 deaths per 100,000, representing a 15% reduction over the 23-year period. Conversely, the oldest age stratum (children aged 2–4 years) demonstrated the most favorable burden profile, with 2023 estimates of 20,000 DALYs per 100,000 population and 200 deaths per 100,000 population, reflecting a 60–70% decline since 2000. Intermediate age groups (1–5 months, 6–11 months, 12–23 months) showed progressive decreases in burden from infancy through early toddlerhood, with 1–5 month-old infants experiencing 140,000 DALYs and 1,500 deaths per 100,000 in 2023 (approximately 30% decline) and 12–23 month-old children experiencing 50,000 DALYs and 500 deaths per 100,000 (approximately 65% decline). Incidence and prevalence trajectories paralleled mortality and DALY trends, declining from 300,000 to 350,000 cases per 100,000 in infants aged less than 28 days to 100,000–150,000 cases per 100,000 in children aged 2–4 years (Fig. 2).

**Fig. 3:**
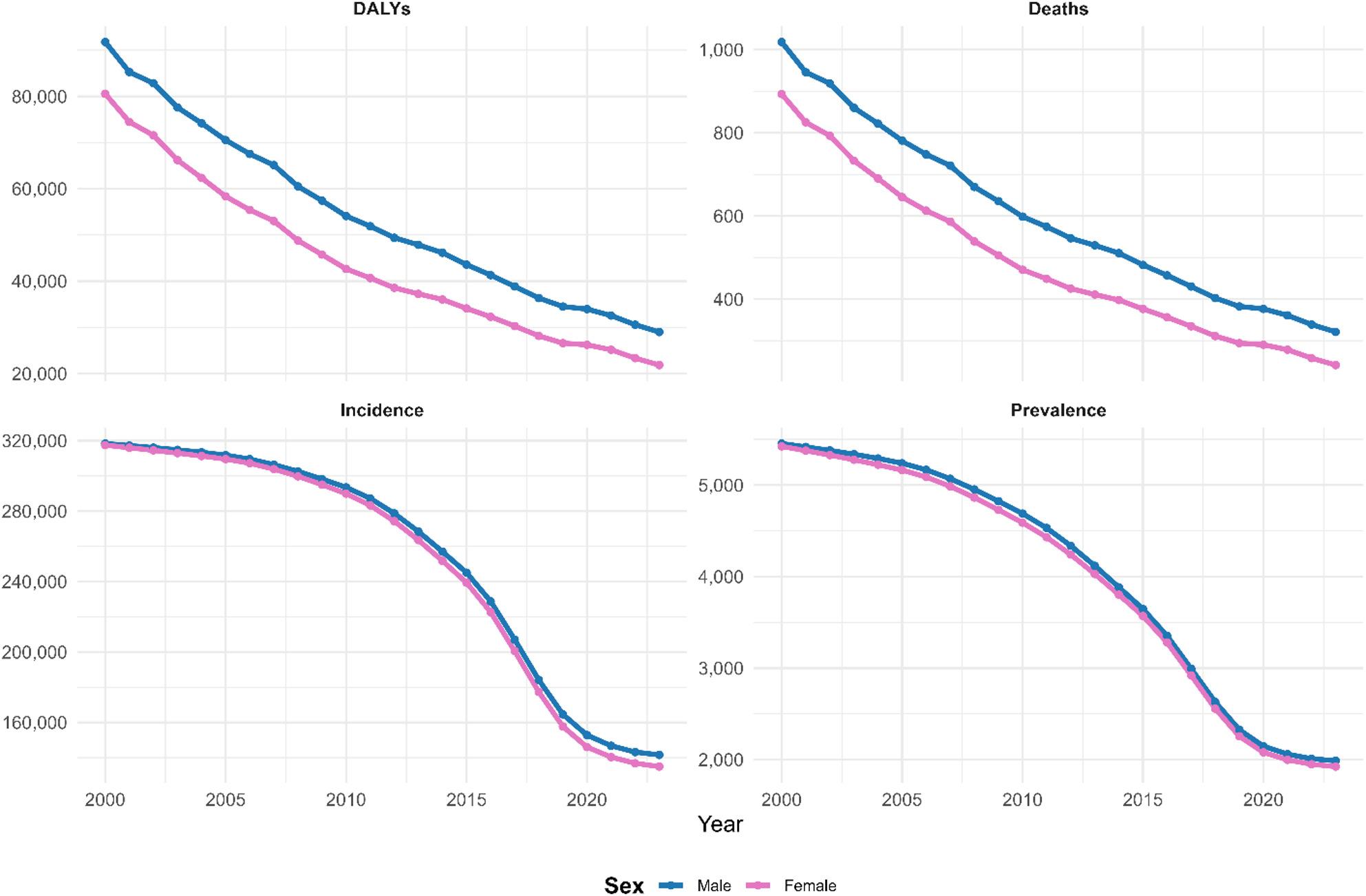
Sex-based epidemiological disparities in diarrhoeal disease among under-five children in East Africa (2000–2023)

Figure 3 reveals that the diarrheal disease burden demonstrated consistent sex-based disparities, with males experiencing a substantially higher absolute burden across all four epidemiological metrics. In 2023, males experienced 30,000 DALYs per 100,000 and approximately 350 deaths per 100,000, compared to females with 22,000 DALYs per 100,000 and 280 deaths per 100,000, representing 36% and 25% higher burdens, respectively. The incidence rates in 2023 converged substantially between sexes at approximately 145,000–150,000 cases per 100,000, compared with 2000 estimates of 315,000 cases per 100,000 for both sexes, indicating a 52–54% reduction over the study period with parallel decline trajectories. Prevalence similarly demonstrated convergence in 2023 at approximately 1,900–2,000 per 100,000 (compared to 5,000–5,500 per 100,000 in 2000), representing a 61–65% decline with minimal sex-based differentials by the end of the study period. However, mortality and DALY trends revealed sustained sex-based disparities throughout 2000–2023, with males maintaining a consistent 20–30% higher mortality and morbidity burden, suggesting that incidence and prevalence declines have been comparable between sexes.

As shown in Table 2, consistent annual declines in diarrheal disease mortality, DALYs, incidence, and prevalence among children under five across most East African countries between 2000 and 2023. The steepest reductions in mortality and DALYs were observed in Rwanda, Ethiopia, Tanzania, and Burundi, with AAPCs exceeding − 7% per year, while more modest declines were evident in Madagascar and Somalia. In contrast, South Sudan demonstrated minimal or non-significant annual change in mortality and DALYs, indicating stalled progress relative to regional trends. Overall, incidence and prevalence declined more slowly than mortality and DALYs, highlighting persistent transmission despite improvements in survival.

**Table 2:** Average annual percentage change (AAPC) in diarrheal disease burden among children under five years in East Africa, 2000–2023

location	DALYs	Deaths	Incidence	Prevalence
Burundi	-7.75 (-8.22 to -7.29)	-7.77 (-8.23 to -7.30)	-5.70 (-6.89 to -4.48)	-6.85 (-8.08 to -5.60)
Comoros	-5.88 (-6.56 to -5.19)	-5.86 (-6.57 to -5.15)	-6.12 (-6.91 to -5.33)	-6.96 (-7.77 to -6.15)
Djibouti	-6.16 (-6.56 to -5.76)	-6.18 (-6.57 to -5.78)	-4.07 (-4.72 to -3.41)	-5.00 (-5.71 to -4.28)
Eritrea	-5.29 (-5.47 to -5.10)	-5.28 (-5.46 to -5.09)	-5.65 (-6.38 to -4.90)	-6.52 (-7.19 to -5.83)
Ethiopia	-7.25 (-7.65 to -6.85)	-7.27 (-7.69 to -6.85)	-5.94 (-6.84 to -5.02)	-6.54 (-7.41 to -5.66)
Kenya	-6.82 (-7.01 to -6.64)	-6.87 (-7.05 to -6.69)	-4.06 (-4.66 to -3.45)	-4.42 (-5.04 to -3.79)
Madagascar	-4.09 (-4.69 to -3.47)	-4.08 (-4.69 to -3.47)	-3.05 (-3.75 to -2.35)	-4.01 (-4.76 to -3.26)
Rwanda	-8.85 (-9.36 to -8.33)	-8.88 (-9.42 to -8.34)	-5.79 (-6.65 to -4.93)	-6.84 (-7.71 to -5.96)
Somalia	-4.77 (-4.92 to -4.63)	-4.76 (-4.91 to -4.62)	-5.51 (-6.40 to -4.62)	-6.34 (-7.21 to -5.47)
South Sudan	-0.27 (-0.66 to 0.11)	-0.22 (-0.62 to 0.18)	-3.40 (-4.17 to -2.62)	-4.15 (-5.02 to -3.27)
Uganda	-6.65 (-7.25 to -6.03)	-6.62 (-7.23 to -6.00)	-6.25 (-7.43 to -5.05)	-7.15 (-8.33 to -5.96)
United Republic of Tanzania	-7.57 (-8.27 to -6.87)	-7.59 (-8.28 to -6.89)	-6.16 (-7.21 to -5.11)	-6.98 (-8.01 to -5.93)

**Fig. 4:**
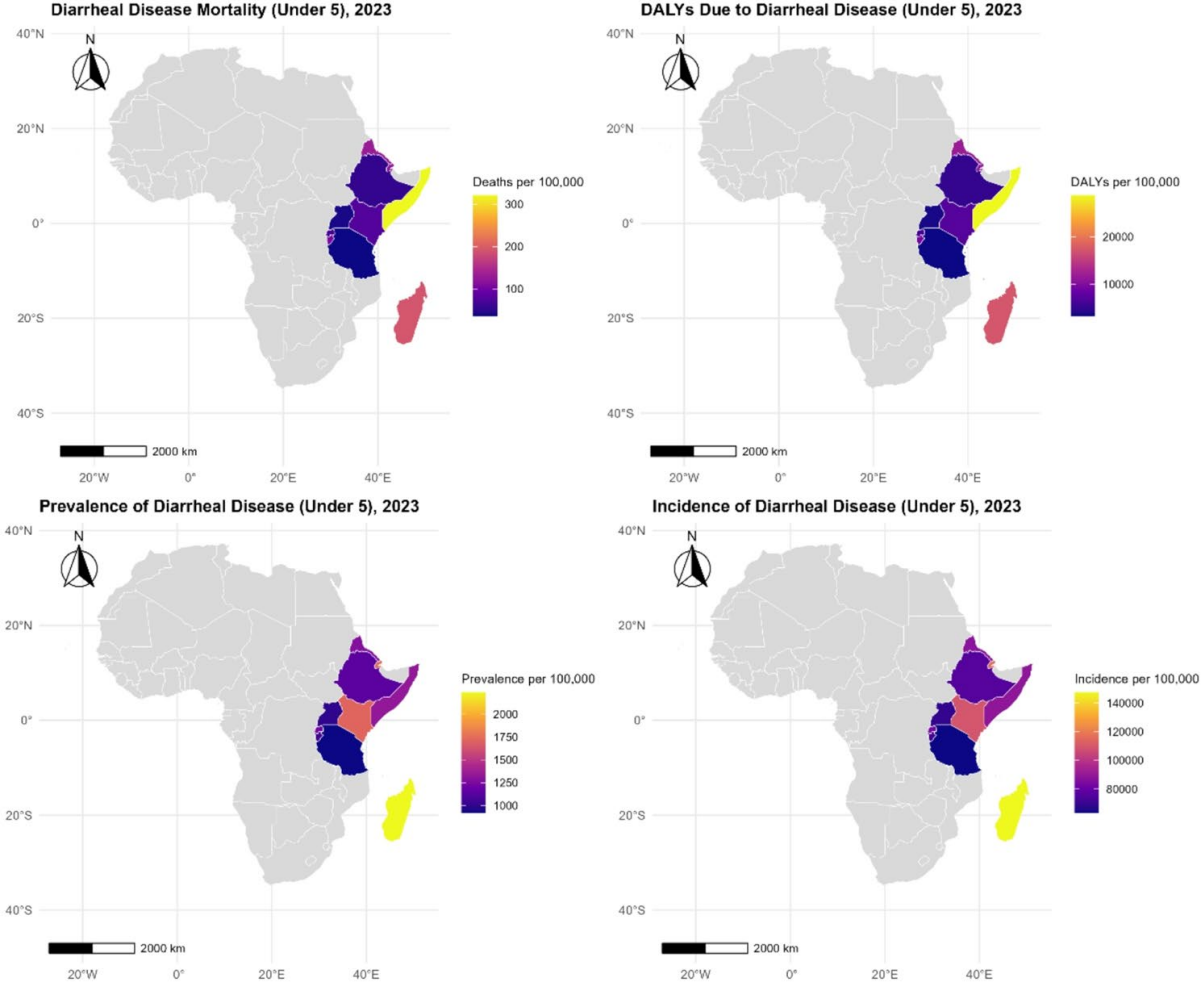
Geographic variation in diarrhoeal disease burden across East African countries (2023)

Figure 4 illustrates marked geographic heterogeneity in the burden of diarrheal diseases among children under five across Africa in 2023, with East African countries exhibiting substantially higher mortality, DALY, prevalence, and incidence rates compared to much of the continent. The highest burden is concentrated in the Horn of Africa, particularly Somalia and South Sudan, while comparatively lower rates are observed in countries such as Tanzania, Uganda, and Rwanda. The grey shading across other African countries reflects the absence of country-specific data in this analysis and emphasizes the regional focus on East Africa. Overall, the maps highlight persistent spatial inequities in diarrheal disease burden, closely aligned with disparities in health system capacity, WASH infrastructure, conflict exposure, and climate vulnerability.

WASH deficiencies accounted for a substantial diarrheal disease burden across East African countries in 2023, with marked geographic disparities reflecting inequities in access to fundamental health infrastructure. The total WASH-attributable DALY burden ranged from a minimum of 3,057 per 100,000 in Tanzania (95% UI 2,003.7–4,382.9) and 3,477 per 100,000 in Uganda (95% UI 1,583.2–6,369.9) to a maximum of 37,608 per 100,000 in South Sudan (95% UI: 21,869.2–57,511.4) and 27,004 per 100,000 in Somalia (95% UI: 15,569.1–40,487.7), representing a 12-fold difference between the best and worst performing countries. Unsafe water sources consistently contributed the largest share of the WASH-attributable burden across all countries, ranging from 2,427 per 100,000 (Tanzania) to 30,908 per 100,000 (South Sudan). Unsafe sanitation represented the second largest contributor, ranging from 2,049 per 100,000 (Tanzania) to 26,200 per 100,000 (South Sudan), while limited access to handwashing facilities contributed the smallest share (800–12,005 per 100,000) but remained a consistent burden driver. Madagascar demonstrated a notably high WASH-attributable burden (16,223 per 100,000), driven primarily by unsafe water (13,054 per 100,000) (Table 3).

**Table 3:** DALY rates (per 100 000 population) attributable to selected WASH-related risk factors for diarrheal diseases among children under five years (< 5 years) in East Africa, 2023 (GBD 2023)

location	Unsafe water, sanitation, and handwashing	Unsafe sanitation	No access to handwashing facility	Unsafe water source
Burundi	8834.2 (4115.8–15987.2)	6106.7 (2800.0–10764.7)	2837.8 (371.1–6449.5)	7184.0 (2796.0–13791.8)
Comoros	8138.8 (4128.9–13699.2)	5518.4 (2771.0–9003.5)	2261.6 (346.5–4665.4)	6281.3 (2369.1–10903.7)
Djibouti	9897.9 (5143.0–16174.0)	6499.2 (3579.1–10787.0)	2803.1 (396.8–5951.0)	7733.6 (3159.9–13329.2)
Eritrea	11016.3 (5676.1–18209.3)	7601.9 (4184.1–12606.0)	3401.9 (538.6–6732.9)	8786.2 (3562.4–14946.4)
Ethiopia	4660.6 (2555.2–7882.2)	3296.7 (1801.5–5559.7)	1527.1 (221.0–3056.3)	3749.4 (1601.9–6640.1)
Kenya	6556.5 (4313.8–9455.2)	4322.2 (2974.1–6112.2)	1893.0 (288.0–3713.9)	5286.6 (2403.5–8012.7)
Madagascar	16222.7 (9158.1–24043.7)	11341.0 (6688.2–17132.1)	4998.3 (706.2–10056.6)	13053.8 (6749.4–20184.6)
Rwanda	5755.7 (2949.2–9554.7)	3862.6 (2076.0–6623.5)	1848.4 (272.7–3610.0)	4572.3 (1723.5–7831.8)
Somalia	27003.7 (15569.1–40487.7)	18421.1 (10503.5–28321.0)	8159.4 (1232.9–15596.3)	21960.4 (10082.7–35375.1)
South Sudan	37608.3 (21869.2–57511.4)	26200.2 (15006.5–40016.1)	12004.6 (1934.6–23049.1)	30907.5 (14354.8–49824.6)
Uganda	3477.4 (1583.2–6369.9)	2382.8 (1125.5–4453.0)	1095.2 (129.4–2433.5)	2754.7 (974.3–5055.9)
Tanzania	3057.0 (2003.7–4382.9)	2049.1 (1377.3–2962.8)	800.3 (141.3–1588.9)	2427.5 (1223.9–3748.6)

## Discussion

This comprehensive analysis of diarrheal disease burden among children under five years of age in East Africa from 2000 to 2023 reveals substantial but highly heterogeneous progress toward mortality reduction, with profound disparities in disease burden persisting across countries despite two decades of global health investment. Mortality declined by 60–87% in countries achieving substantial progress—Tanzania (84%), Rwanda (88%), Uganda (82%), and Ethiopia (86%)— along with corresponding reductions in disability-adjusted life-years ranging from 80% to 87%, whereas Somalia, South Sudan, and Madagascar sustained persistently elevated mortality (320–443 per 100,000) and DALYs (17,200–39,700 per 100,000), representing minimal improvement over the study period. This 12-fold difference in 2023 mortality rates between the best-performing (Tanzania at 36 per 100,000) and worst-performing (South Sudan at 443 per 100,000) countries underscores fundamental inequities in access to preventive and curative interventions, with disease burden concentrated disproportionately in conflict-affected and climate-vulnerable regions of the Horn of Africa [40]. The parallel decline in mortality and DALYs across improving countries indicates that reductions in fatal outcomes constituted the primary mechanism driving overall burden mitigation, likely attributable to the expanded coverage of oral rehydration therapy, zinc supplementation, rotavirus vaccination, and improved case management capacity [3].​​.

Comparatively, East African progress aligns with broader global diarrheal disease mortality reductions—the 60% global decline from 1990 to 2021 documented in the Global Burden of Disease 2021 study mirrors the reductions observed in top-performing East African countries [4]. However, regional disparities within East Africa exceed those documented between global income strata, with South Sudan’s 15% mortality decline representing substantially worse performance than the 79% reduction in children under five years globally [4]. The concentrated burden in Somalia and South Sudan reflects epidemiological patterns observed in other conflict-affected and fragile states globally, where protracted crises disrupt essential health services, displace populations in overcrowded settlements with minimal water and sanitation infrastructure, and create conditions conducive to sustained diarrheal disease transmission [5, 40]. Evidence from the Vaccine Impact on Diarrhea in Africa (VIDA) study demonstrated that sites achieving substantial mortality reductions between 2007 and 2011 (Global Enteric Multicenter Study period) and 2015–2018 (VIDA study period) primarily contributed to reduced childhood wasting (27%), increased rotavirus vaccine coverage (23%), zinc supplementation for diarrhea treatment (12%), and oral rehydration therapy (10%) [3, 41]. These findings corroborate our observed age-stratified patterns, wherein neonates (< 28 days) demonstrated minimal burden reduction (15% over 23 years) compared to older children (60–80% decline in children aged 2–4 years), suggesting that mortality reduction interventions, particularly rotavirus vaccination introduced at 6–12 weeks, have disproportionately benefited older infants, while neonatal diarrheal disease prevention remains insufficiently addressed through maternal and neonatal interventions [4, 6].

Rotavirus vaccine introduction in East Africa occurred after the neonatal period, with schedules initiating at 6–14 weeks of age (Rwanda, Tanzania and Kenya), limiting direct protection among neonates [42–44]. Although these introductions were associated with substantial mortality reductions in older children, neonatal diarrheal mortality declined by only ~ 15% between 2000 and 2023 across both vaccinating and non-vaccinating countries, indicating that delayed vaccine timing alone does not explain the persistently high neonatal burden [42, 44]. Evidence from post-vaccine surveillance suggests that neonatal diarrhea is disproportionately driven by bacterial pathogens rather than rotavirus [44], while limited postnatal care access, high home-delivery rates, delayed care-seeking, and diagnostic overlap with neonatal sepsis further constrain effective early management, underscoring the need for neonatal-specific preventive and care strategies.

The marked intercountry variation in burden reduction correlates strongly with WASH infrastructure development and health system strengthening investments over the study period [2, 40]. Countries achieving substantial mortality reductions—Rwanda, Tanzania, Uganda, and Ethiopia—simultaneously expanded water coverage from 45% to 65% in 2000 to 75% to 85% by 2023 and improved sanitation coverage from 30% to 50% to 60% to 75% [3]. Quantitatively, WASH-attributable DALYs in 2023 ranged from 3,057 per 100,000 in Tanzania to 37,608 per 100,000 in South Sudan, with unsafe water sources contributing 70–80% of this burden (2,427–30,908 per 100,000) and unsafe sanitation contributing 60–70% (2,049–26,200 per 100,000). These findings corroborate propensity score-matched analyses demonstrating that improved water access reduces diarrhea prevalence by 24–35% and improved sanitation by 28–41% in rural low- and middle-income settings [5]. However, recent evidence from the Democratic Republic of Congo reveals that infrastructure improvements alone prove insufficient without concurrent behavior change and integrated nutrition interventions, as community-driven WASH interventions increased access, but failed to reduce diarrhea prevalence or growth faltering [45]. This suggests that sustained burden reduction requires multi-sectoral approaches that couple infrastructure development with health education, community mobilization, and treatment access [6].​​.

Rwanda exemplifies successful integrated programming, achieving 88% mortality reduction through coordinated investments in community health workers, rotavirus vaccination, and the WASH infrastructure. Between 2008 and 2011, Rwanda implemented nationwide integrated community case management (iCCM) training for community health workers in all 15,000 villages, equipping them for empirical diagnosis and treatment of pneumonia, diarrhea, and malaria, along with malnutrition surveillance and comprehensive reporting [10, 46]. Post-implementation analysis documented significant increases in community-based treatment coverage for diarrhea and pneumonia, alongside substantial declines in under-five mortality and facility overcrowding [10]. Ethiopia’s Health Extension Program similarly achieved substantial burden reduction through household water disinfection using sodium hypochlorite and handwashing promotion, demonstrating a 36–41% diarrhea incidence reduction in rural settings through low-cost, behavior-change interventions delivered via existing primary healthcare platforms [16]. Rotavirus vaccine introduction across East Africa represented a critical inflection point, with 31 of the 47 African countries achieving introduction by 2016 at 77% coverage, reducing the proportion of pediatric diarrhea hospitalizations attributable to rotavirus from 39% to 26% post-introduction [9]. However, vaccine effectiveness remains suboptimal in African settings (60% in Ghana) [47] compared to high-income countries (85–95%) [10], which is attributable to enteropathy, malnutrition, concurrent enteric infections, and maternal antibody interference [48].

The persistently higher diarrheal mortality and DALYs among males (25–36%), despite convergent incidence and prevalence between sexes, represent an important equity concern [49]. Similar male excess mortality has been documented for other childhood infections, including pneumonia, suggesting that factors beyond pathogen exposure—such as biological vulnerability and differences in disease severity or treatment effectiveness—may contribute [49, 50]. These findings highlight the importance of sex-disaggregated surveillance and targeted evaluation of care-seeking behavior and quality of care to identify and address avoidable sex-based disparities in child survival.

Progress toward Sustainable Development Goal 3.2—achieving under-five mortality of 25 per 1,000 live births by 2030—remains substantially off-track in East Africa, with only eight of 54 African countries reaching this threshold by 2023 [15, 51]. Somalia and South Sudan’s persistently elevated burden reflects the devastating health consequences of protracted conflict, which systematically dismantled health infrastructure, disrupted vaccine delivery, displaced populations, and constrained WASH access [22, 52]. Droughts in Sub-Saharan Africa have tripled between 1970 and 1979 and 2010–2019, intensifying water scarcity and forcing reliance on contaminated sources. Climate change is projected to result in approximately 250,000 additional deaths annually—beyond baseline mortality projections—between 2030 and 2050 in low- and middle-income countries, with diarrheal diseases accounting for an estimated 48,000 deaths (approximately 19%) [53, 54]. In East Africa, countries in the Horn of Africa—including Somalia, South Sudan, Ethiopia, and parts of Kenya—are expected to experience the greatest climate-related increases in diarrheal risk due to drought, flooding-related water contamination, and food insecurity, although country-specific quantitative projections remain limited [55].

### Study limitations

This study’s reliance on the Global Burden of Disease model estimates introduces inherent limitations, including uncertainty intervals reflecting data sparsity, potential misclassification of diarrheal deaths in settings with incomplete vital registration, and limited granularity for within-country spatial heterogeneity beyond national aggregates. The GBD framework employs DisMod-MR 2.1, a Bayesian meta-regression model that borrows strength across geographies and periods to generate estimates for data-sparse contexts, introducing model-based assumptions that may not fully capture local epidemiological realities. Validation against independent data sources, including Demographic and Health Surveys, Multiple Indicator Cluster Surveys, and facility-based surveillance systems, would strengthen confidence in estimates, particularly for Somalia and South Sudan, where data availability remains severely constrained. Risk-attributable burden estimates rely on a comparative risk assessment methodology integrating exposure data, relative risks from meta-analyses, and population-attributable fractions, which assume causality based on observational evidence and may overestimate or underestimate attributable burden depending on residual confounding, measurement error, and effect modification across contexts. The inability to directly assess intervention coverage, quality of care, or specific policy implementation timelines limits the mechanistic interpretation of observed mortality trends, necessitating complementary implementation research and program evaluation to identify transferable lessons from high-performance countries. Despite these limitations, the standardized GBD methodology permits robust cross-country and temporal comparisons, providing essential evidence for policymakers, donors, and implementing partners to prioritize interventions and allocate resources to the highest-burden populations.

## Conclusion

East Africa has achieved substantial but profoundly inequitable progress in diarrheal disease mortality reduction among children under five between 2000 and 2023, with gains concentrated in countries that expanded the WASH infrastructure, introduced rotavirus vaccination, strengthened community health worker systems, and maintained functional primary healthcare platforms. The 12-fold variation in mortality rates between the best- and worst-performing countries in 2023 reflects structural inequities in access to water, sanitation, nutrition, and healthcare that transcend technical interventions and require sustained political commitment, equitable financing, and regional collaboration to address the underlying determinants of health. Achieving SDG 3.2 by 2030, necessitates the emergency acceleration of progress in Somalia, South Sudan, and Madagascar through targeted humanitarian support, conflict resolution, climate adaptation investment, and health system reconstruction, alongside sustained investment in high-performing countries to prevent backsliding and address the remaining burden among neonates and marginalized populations. The lessons from Rwanda, Tanzania, Uganda, and Ethiopia, demonstrating that sustained mortality reductions remain achievable in resource-constrained settings through community-based platforms, WASH investment, and vaccine introduction, offer transferable models for the region, provided that interventions are adapted to local epidemiological, social, and political contexts and implemented with fidelity, equity, and accountability.

## Data Availability

All data analyzed in this study are publicly available from the Global Burden of Disease 2023 (GBD 2023) Results Tool, hosted by the Institute for Health Metrics and Evaluation (IHME) at https://www.healthdata.org/research-analysis/about-gbd. No primary data were collected, and no individual-level data were used.

## Abbreviations

CHW: Community Health Worker
GBD: Global Burden of Disease
IDP: Internally Displaced Person
ORS: Oral Rehydration Solution
SDG: Sustainable Development Goal
WASH: Water, Sanitation, and Hygiene
WHO: World Health Organization
